# Delayed Resolution of Hoarseness following Bronchial Artery Embolization for a Bronchial Artery Aneurysm: A Case Report

**DOI:** 10.70352/scrj.cr.26-0528

**Published:** 2026-09-30

**Authors:** Hideomi Ichinokawa, Kaori Shimizu, Hiroshi Ikeuchi, Yukio Watanabe, Kazuya Takamochi, Kenji Suzuki

**Affiliations:** 1 Department of General Thoracic Surgery, Juntendo University Shizuoka Hospital, Izunokuni, Shizuoka, Japan; 2 Department of General Thoracic Surgery, Juntendo University Hospital, Tokyo, Japan

**Keywords:** bronchial artery aneurysm, recurrent laryngeal nerve palsy, hoarseness, bronchial artery embolization, endovascular treatment

## Abstract

**INTRODUCTION:**

Bronchial artery aneurysms (BAAs) are rare vascular lesions that require prompt treatment because of the risk of rupture, which can be life-threatening regardless of their size. Hoarseness caused by recurrent laryngeal nerve (RLN) palsy secondary to BAA is extremely uncommon, and delayed clinical improvement after treatment has rarely been reported. Herein, we report a case of a mediastinal BAA presenting with hoarseness that gradually resolved following bronchial artery embolization (BAE).

**CASE PRESENTATION:**

A 73-year-old woman presented with hoarseness persisting for 8 months. Laryngoscopic examination revealed left vocal cord paralysis, and contrast-enhanced CT demonstrated a 36-mm mediastinal BAA associated with markedly dilated and tortuous bronchial arteries and bronchiectatic changes in the left lower lobe. The aneurysm was deemed to be responsible for compression of the left RLN. BAE was subsequently conducted using coil embolization of both the aneurysm sac and the feeding artery. The postoperative course was uneventful, and the patient was discharged on POD 3. Follow-up CT at 7 months revealed a reduction in aneurysm diameter from 36 to 25 mm, with near-complete disappearance of intra-aneurysmal blood flow. Hoarseness gradually improved during follow-up and completely resolved 17 months following embolization.

**CONCLUSIONS:**

RLN palsy caused by BAA is rare. Postoperative laryngoscopic confirmation of vocal cord motion was unavailable. However, this case demonstrates that hoarseness may gradually resolve long after endovascular treatment. Progressive aneurysm shrinkage following embolization may have gradually relieved compression of the RLN, thereby contributing to the delayed improvement in hoarseness. Therefore, long-term follow-up should be considered in patients with BAA presenting with hoarseness.

## Abbreviations

BAA
: bronchial artery aneurysm
BAE
: bronchial artery embolization
Fr
: French (catheter size)
RLN
: recurrent laryngeal nerve
TEVAR
: thoracic endovascular aortic repair

## INTRODUCTION

BAAs are rare vascular lesions, accounting for only a very small proportion of bronchial arterial abnormalities; however, they require prompt treatment as rupture can be life-threatening regardless of their size.^1,2)^ Endovascular treatment has increasingly replaced surgical repair as the preferred therapeutic strategy because of its minimally invasive nature and favorable outcomes.^3–5)^ To the best of our knowledge, only a few cases of RLN palsy secondary to BAA with improvement in hoarseness after treatment have been reported,^6,7)^ and the time course and mechanisms of clinical recovery remain poorly understood.

Herein, we describe a case of a mediastinal BAA presenting with hoarseness because of left RLN palsy, successfully treated with BAE, achieving aneurysm shrinkage and gradual resolution of hoarseness during follow-up. In previously reported cases, improvement in hoarseness occurred within several days to 3 months after treatment, whereas our patient experienced complete resolution of hoarseness 17 months after embolization.

## CASE PRESENTATION

The patient was a 73-year-old woman with no relevant medical history and no history of smoking. She had experienced hoarseness for 8 months and eventually visited an otolaryngology specialist because of persistent symptoms. Pharyngeal endoscopy revealed fixation of the left vocal cord, resulting in a diagnosis of left RLN paralysis. A plain CT scan of the neck and chest was conducted to investigate the cause, revealing a 36-mm mass lesion protruding to the left in the left upper mediastinum. She was consequently referred to our hospital for further examination and treatment.

Blood tests revealed no remarkable abnormalities. Because the lesion was initially suspected to represent a mediastinal mass, contrast-enhanced CT was subsequently performed at our hospital, revealing marked dilation and tortuosity of the bronchial arteries branching from the aortic arch and draining into an aneurysm measuring 36 mm in diameter (**Fig. 1A**–**1C**). Furthermore, dilation and tortuosity were observed in the peripheral bronchial arteries, branching from the descending thoracic aorta. In addition, consistent with bronchiectasis, cystic bronchiectasis, centrilobular granular opacities, and nodular opacities were observed, predominantly in the left lower lobe, findings compatible with chronic inflammatory airway disease (**Fig. 1D**, **1E**). Bronchiectasis had not been previously diagnosed before referral. Based on the above findings, BAA associated with bronchiectasis compressing the left RLN and causing hoarseness was diagnosed. Because BAAs carry a substantial risk of rupture regardless of their size, treatment was undertaken to prevent rupture and potentially relieve RLN compression.

**Fig. 1:**
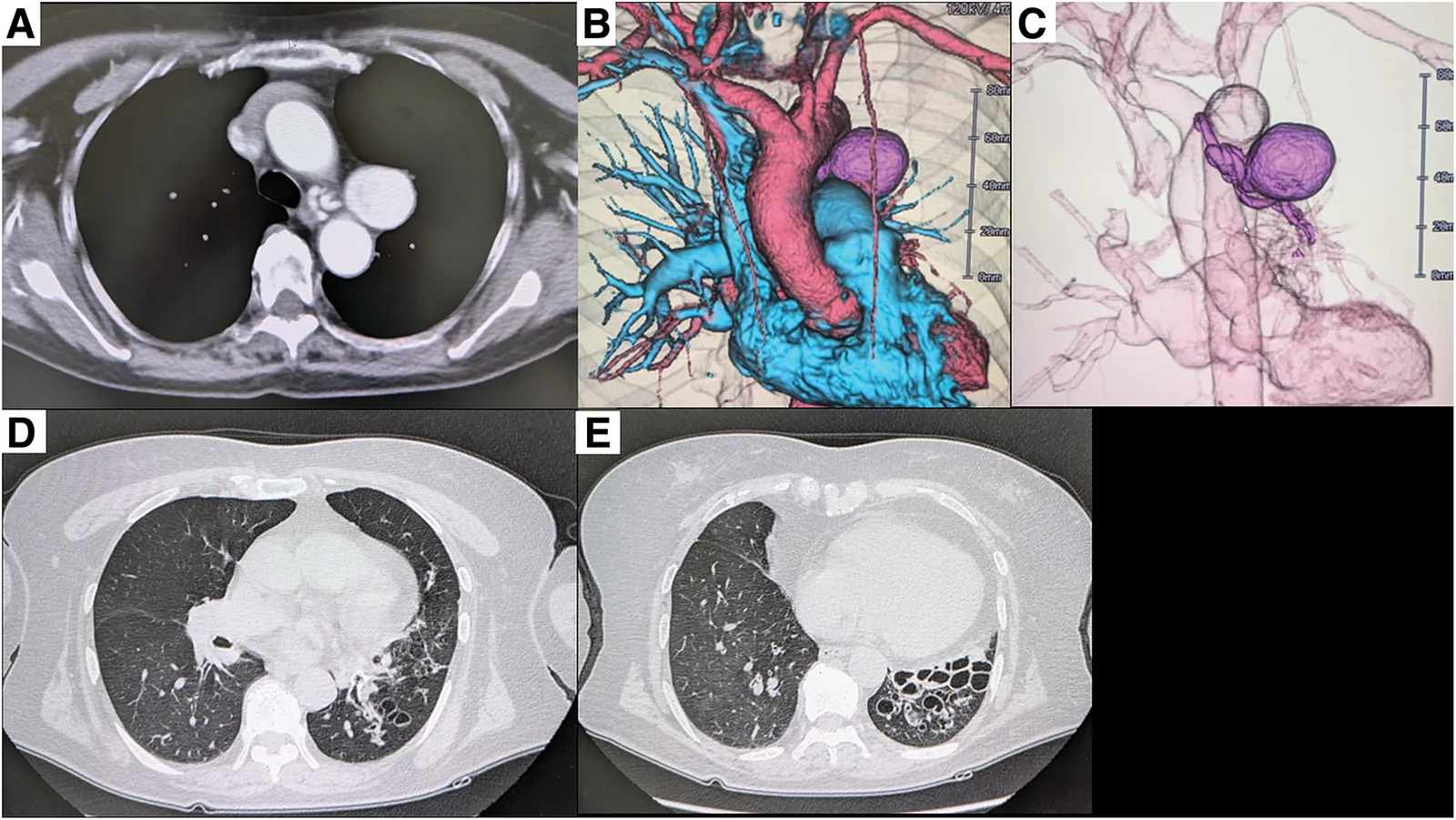
Findings of preoperative contrast-enhanced CT. (**A**) Axial CT image showing a 36-mm mediastinal BAA. (**B**) 3D-CTA reconstructed from arterial- and venous-phase images. The BAA is highlighted in purple, demonstrating its anatomical relationship with the surrounding mediastinal structures. (**C**) 3D-CTA reconstructed following removal of the arterial and venous structures. The BAA is highlighted in purple, allowing clear visualization of its morphology. (**D**) Lung-window CT demonstrating cystic bronchiectasis, centrilobular nodules, and bronchial wall thickening predominantly in the left lower lobe, consistent with chronic bronchial inflammation. (**E**) Lung-window CT at a lower level showing extensive cystic bronchiectatic changes in the left lower lobe. BAA, bronchial artery aneurysm

BAE proceeded as follows: The right femoral artery was punctured using the Seldinger technique, and a 4-Fr long sheath was placed. Using a bronchial artery catheter, the left bronchial artery was selected, and a tortuous afferent vessel and peripheral aneurysm were visualized (**Fig. 2A**). Subsequently, a 1.9-Fr microcatheter (Carnelian MARVEL non-taper 160 cm, Tokai Medical Products, Aichi, Japan) was advanced into the aneurysm under microguidewire guidance; 9 and 5 coils were placed in the aneurysm and afferent vessel, respectively, for embolization. Post-embolization contrast-enhanced imaging confirmed a good embolization effect with disappearance of blood flow within the aneurysm. As such, no additional liquid embolizing material, such as N-butyl-2-cyanoacrylate, was used (**Fig. 2B**).

**Fig. 2:**
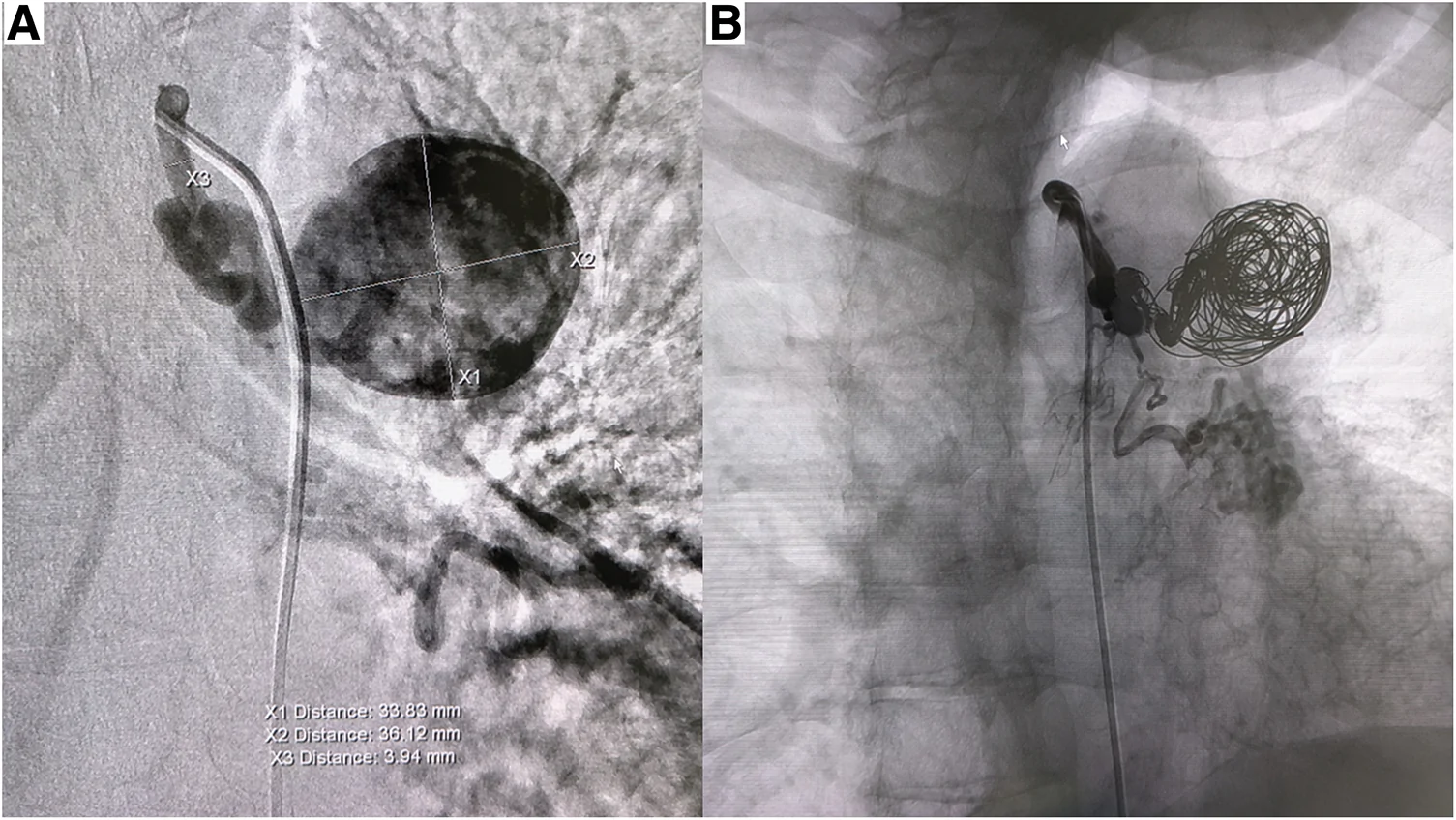
Angiographic findings during BAE. (**A**) Selective bronchial arteriography revealing a tortuous feeding artery and aneurysm. (**B**) Completion angiography showing complete exclusion of the aneurysm after coil embolization. BAE, bronchial artery embolization

The postoperative course was uneventful, and the patient was discharged on the third postoperative day without complications. The aneurysm diameter remained 36 mm at 1 month after embolization (**Fig. 3A**) and decreased to 30 mm at 4 months (**Fig. 3B**). At 7 months after embolization, contrast-enhanced CT revealed a further decrease in aneurysm diameter to 25 mm, with near-complete disappearance of intra-aneurysmal blood flow (**Fig. 3C**). The aneurysm remained reduced in size at 17 months (**Fig. 3D**) and 24 months (**Fig. 3E**). Hoarseness gradually improved during outpatient follow-up, disappearing by 17 months after embolization. Postoperative laryngoscopic examination was not performed because the patient’s symptoms had resolved. The patient continues to undergo annual chest CT and chest X-ray examinations, and no recurrence has been observed during 24 months of follow-up.

**Fig. 3:**
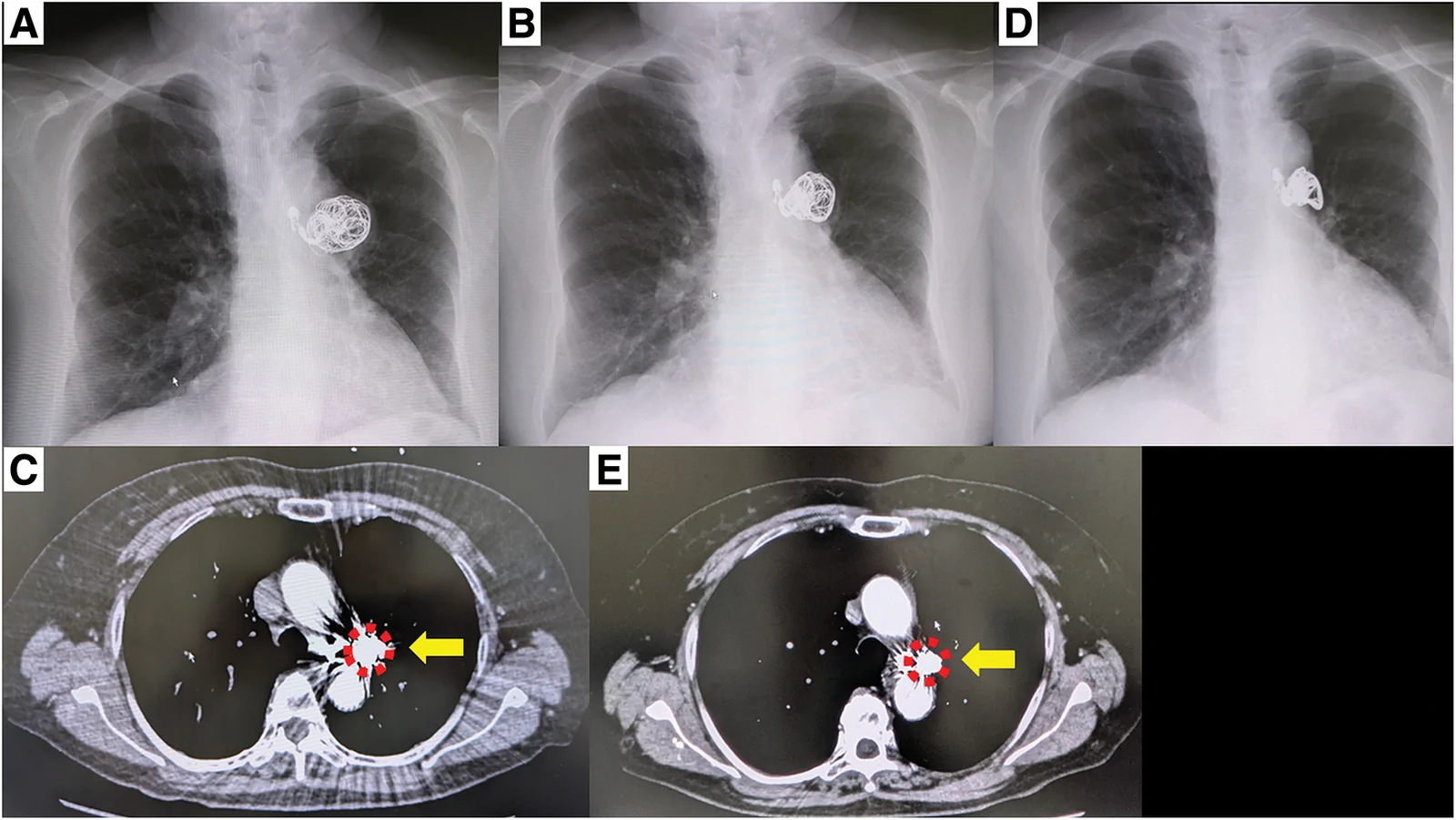
Serial chest radiographs and CT images demonstrating progressive aneurysm shrinkage following BAE. (**A**) Chest radiograph obtained 1 month following embolization showing a 36-mm aneurysm, with no significant change in size compared with the preoperative image. (**B**) Chest radiograph obtained 4 months after embolization demonstrating reduction of the aneurysm to 30 mm. (**C**) Contrast-enhanced CT conducted 7 months after embolization showing a decrease in aneurysm diameter to 25 mm, with near-complete disappearance of intra-aneurysmal blood flow. The dashed outline indicates the estimated contour of the aneurysm, whereas arrow indicates the aneurysm location despite coil-related artifacts. (**D**) Chest radiograph obtained 17 months after embolization demonstrating sustained aneurysm shrinkage, with the aneurysm measuring approximately 25 mm. (**E**) Contrast-enhanced CT conducted 24 months after embolization confirming persistent aneurysm regression, with the aneurysm diameter remaining at 25 mm and no evidence of recanalization. The dashed outline indicates the estimated contour of the aneurysm, whereas arrow indicates the aneurysm location despite coil-related artifacts. BAE, bronchial artery embolization

A timeline of the patient’s clinical course is presented in **Fig. 4**.

**Fig. 4:**
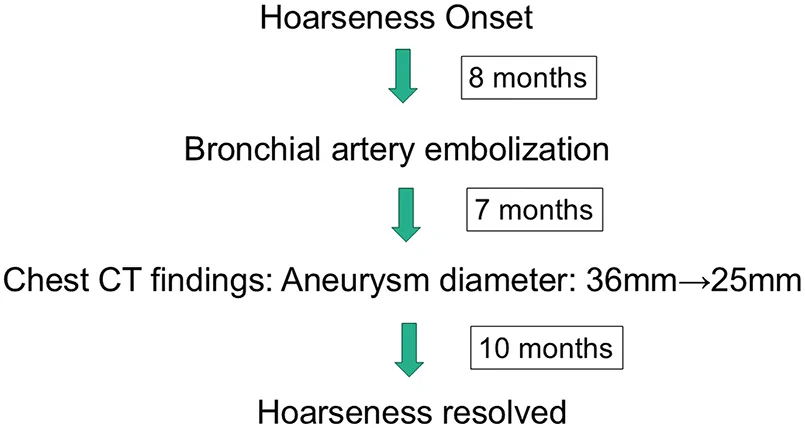
Clinical timeline of the patient. The patient experienced hoarseness for 8 months before treatment. BAE achieved gradual aneurysm shrinkage, with complete resolution of hoarseness 17 months later. BAE, bronchial artery embolization

## DISCUSSION

Depending on their site of origin, BAAs are classified into mediastinal and pulmonary types. Mediastinal BAAs may present with a wide spectrum of symptoms, depending on rupture status, aneurysm size, and relationship to adjacent structures.^2,8)^ The present case presented with hoarseness as the chief complaint.

Typical causes of hoarseness include structural abnormalities of the vocal cords, such as vocal cord polyps, polypoid degeneration, and laryngeal malignancies, in addition to RLN palsy. Common causes of RLN palsy include cardiovascular disorders and intrathoracic malignancies.^9)^ RLN palsy associated with cardiovascular disease is commonly referred to as Ortner’s syndrome or cardiovocal syndrome; it has been reported in association with thoracic aortic aneurysms, pulmonary hypertension, mitral valve disease, and other cardiovascular abnormalities.^10)^ Although BAA is not traditionally included within this entity, the underlying mechanism, i.e., extrinsic compression of the left RLN by an enlarged vascular structure, is essentially the same. Therefore, the present case may be considered a rare variant of cardiovocal syndrome.

In contrast, RLN palsy caused by BAA is extremely rare, with only a few previously reported cases describing improvement in hoarseness after treatment.^6,7)^

Up to 40% of patients with unilateral vocal cord paralysis are asymptomatic at diagnosis, and underlying disease-related symptoms may delay diagnosis.^9)^ Recovery from RLN palsy is influenced by the severity and duration of nerve injury. In the present case, although hoarseness had persisted for 8 months prior to treatment, it gradually improved and completely resolved within 17 months of BAE treatment. Previous reports of BAA-associated RLN palsy have described improvement in hoarseness occurring within days to 3 months after treatment.^6,7)^ In particular, Mitomo et al. reported improvement in hoarseness within 3 months of BAE.^7)^ In contrast, complete resolution of hoarseness occurred much later in our patient, at 17 months after embolization.

One possible explanation for the delayed clinical improvement observed in the present case is the gradual relief of chronic compression of the left RLN following BAE. After successful embolization, the aneurysm progressively decreased from 36 to 25 mm during follow-up, suggesting that the mass effect on the RLN was relieved gradually rather than immediately. Chronic nerve compression is known to induce focal ischemia, impaired axonal transport, and segmental demyelination, all of which may contribute to conduction block and vocal cord paralysis.^11)^ As mechanical compression resolves, functional recovery depends on biological processes such as remyelination, restoration of axonal transport, and, in more severely injured nerves, axonal regeneration. These processes generally require several months and may continue for more than a year. Therefore, the gradual reduction in aneurysm size after embolization may have allowed slow neurological recovery, ultimately contributing to the delayed improvement observed in the present case. Chronic bronchial inflammation is considered 1 of the major etiologies of BAA because persistent inflammation promotes hypertrophy, tortuosity, and increased blood flow in the bronchial arteries, ultimately leading to weakening of the arterial wall and aneurysm formation.^3,5,8)^ In the present case, contrast-enhanced CT demonstrated bronchiectatic changes predominantly in the left lower lobe, together with markedly dilated and tortuous bronchial arteries. Although a causal relationship could not be definitively established, these findings suggested that chronic bronchial inflammation associated with bronchiectasis contributed to the development of the BAA.^3,5)^

In recent years, advances in imaging modalities have increased the detection of asymptomatic BAAs. However, mediastinal BAAs are still sometimes misdiagnosed as mediastinal masses, requiring careful evaluation.^2,8)^ Furthermore, no significant difference in aneurysm diameter between ruptured and unruptured cases has been reported, indicating the importance of early intervention after diagnosis.^3,5)^

Treatment options for BAAs include surgical and endovascular approaches. Although surgical repair has traditionally been the mainstay, it is associated with high invasiveness, and endovascular treatment has been adopted as the first-line therapy in recent years.^3,5,6)^ Since 2010, TEVAR, alone or in combination with embolization, has also been reported.^4,12,13)^ However, embolization alone carries a risk of recanalization, with a reported rate of approximately 10%.^3)^ Prior reports have suggested that additional TEVAR is required in some patients when embolization alone is insufficient to completely exclude aneurysmal inflow.^5)^ Additionally, TEVAR alone has been reported to be effective when catheter access to feeding vessels is difficult because of tortuous or narrow feeding arteries.^4,13)^

In the present case, complete embolization of both the aneurysm sac and the feeding artery achieved durable aneurysm exclusion and progressive aneurysm shrinkage, as observed during follow-up. This gradual reduction in aneurysm size was accompanied by complete resolution of hoarseness. Although a causal relationship could not be definitively proven, the temporal association indicated that relief of RLN compression contributed to recovery of vocal cord function. Because postoperative laryngoscopic evaluation was not performed, recovery of vocal cord motion could not be objectively confirmed. Therefore, improvement in hoarseness may reflect recovery of RLN function, compensatory movement of the contralateral vocal cord, or a combination of both. Nevertheless, the close temporal association between aneurysm shrinkage and resolution of hoarseness suggested that gradual relief of RLN compression contributed to the patient’s clinical improvement.

## CONCLUSIONS

Overall, this case suggests that clinical improvement in hoarseness can occur long after successful endovascular treatment, even in patients with hoarseness persisting for several months before intervention. Although postoperative laryngoscopic confirmation of recovery of vocal cord motion was unavailable, the temporal association between progressive aneurysm shrinkage and resolution of hoarseness suggested that gradual relief of RLN compression contributed to the patient’s clinical improvement. Furthermore, this case highlights that BAA should be considered in the differential diagnosis of patients presenting with RLN palsy. This case also supports the use of endovascular treatment as a minimally invasive therapeutic option for appropriately selected patients with mediastinal BAA. Finally, early recognition and appropriate treatment may contribute to favorable long-term clinical outcomes.

## DECLARATIONS

### Availability of data and materials

All data generated and/or analyzed during this study are included in this published article.
